# Do Interactions Between Gut Ecology and Environmental Chemicals Contribute to Obesity and Diabetes?

**DOI:** 10.1289/ehp.1104204

**Published:** 2011-10-31

**Authors:** Suzanne M. Snedeker, Anthony G. Hay

**Affiliations:** 1 Department of Microbiology and the Institute for Comparative and Environmental Toxicology, and; 2 Department of Food Science, Cornell University, Ithaca, New York, USA

**Keywords:** ADME, biotransformation enzymes, diabetes mellitus, diabetogenic, environmental chemicals, gut ecology, metabolic syndrome, microbes, microbiota, obesity, obesogen, obesogenic, persistent organic pollutants, POPs

## Abstract

Background: Gut microbiota are important factors in obesity and diabetes, yet little is known about their role in the toxicodynamics of environmental chemicals, including those recently found to be obesogenic and diabetogenic.

Objectives: We integrated evidence that independently links gut ecology and environmental chemicals to obesity and diabetes, providing a framework for suggesting how these environmental factors may interact with these diseases, and identified future research needs.

Methods: We examined studies with germ-free or antibiotic-treated laboratory animals, and human studies that evaluated how dietary influences and microbial changes affected obesity and diabetes. Strengths and weaknesses of studies evaluating how environmental chemical exposures may affect obesity and diabetes were summarized, and research gaps on how gut ecology may affect the disposition of environmental chemicals were identified.

Results: Mounting evidence indicates that gut microbiota composition affects obesity and diabetes, as does exposure to environmental chemicals. The toxicology and pharmacology literature also suggests that interindividual variations in gut microbiota may affect chemical metabolism via direct activation of chemicals, depletion of metabolites needed for biotransformation, alteration of host biotransformation enzyme activities, changes in enterohepatic circulation, altered bioavailability of environmental chemicals and/or antioxidants from food, and alterations in gut motility and barrier function.

Conclusions: Variations in gut microbiota are likely to affect human toxicodynamics and increase individual exposure to obesogenic and diabetogenic chemicals. Combating the global obesity and diabetes epidemics requires a multifaceted approach that should include greater emphasis on understanding and controlling the impact of interindividual gut microbe variability on the disposition of environmental chemicals in humans.

The prevalence of adult obesity in the United States has risen dramatically over the last three decades from 14.5% (Flegal et al. 1998) to over 33% (Flegal et al. 2010). Medical costs of obesity are estimated to be between $147 and $168 billion per year in the United States and account for up to 16.5% of medical care costs (Cai et al. 2010; Cawley and Meyerhoefer 2010; Finkelstein et al. 2009). Childhood rates of obesity are rising in the United States (Wang et al. 2011) and in many other countries (Wang and Lobstein 2006). Analysis of trends from 1980 to 2008 also show an increase in body mass index (BMI) (Finucane et al. 2011) and diabetes (Danaei et al. 2011) in most geographic areas surveyed world-wide. The origins of the global obesity and diabetes epidemics are multifaceted, with growing evidence that multiple environmental factors contribute to their development. This is exemplified by emerging evidence of the role of gut microbial ecology in obesity and type 1 and type 2 diabetes (Musso et al. 2011; Qin et al. 2010), as well as evidence from human studies and animal models that environmental chemicals may contribute to the development of these diseases (Baillie-Hamilton 2002; Carpenter 2008; Casals-Casas and Desvergne 2011; Grün 2010; Heindel and vom Saal 2009; La Merrill and Birnbaum 2011; Newbold et al. 2008). While interindividual variability in the gut microbiome affects the metabolism of pharmaceuticals (Clayton et al. 2009) and some environmental toxins (Dean and Ma 2007), the impact of gut ecology on the absorption, distribution, metabolism, and excretion (ADME) of xenobiotics, including obesogenic and diabetogenic chemicals, has received little to no attention. We review the scientific evidence that independently links gut ecology and environmental chemicals to obesity and diabetes, providing a framework for suggesting how these environmental factors may interact with these diseases, and identify future research needs to further our understanding of these relationships.

## The Gut Microbiome and Obesity

Gut microbes outnumber human cells by a factor of 10, yet we know surprising little about many of these organisms. The metagenomic sequencing of the human microbiome reveals that there are 3.3 million nonredundant genes, with over 99% of the genes being of bacterial origin (Qin et al. 2010). This gene set is 150-times larger than the human genome. Although certain microbial species appear to be shared by groups of individuals (Arumugam et al. 2011), with > 50 species shared by 90% of the individuals studied, considerable variation occurs both in the types of microbes and in the diversity of microbial functional genes found between individuals (Qin et al. 2010). The notion of a conserved core of functional genes in the microbiome has been supported by studies in monozygote and dizygote twin pairs, though major differences in the abundance of microbes at the phylum level were observed in the microbiome of obese compared with lean twins (Turnbaugh and Gordon 2009; Turnbaugh et al. 2009a). No relationship between specific phyla and obesity has been found in a more recent study, although significant associations between obesity and inferred microbial metabolic activities such as energy harvesting and osmolyte production (based on the presence of genes predicted to encode specific enzymatic activities) were found (Arumugam et al. 2011). This is consistent with the observations of Calvani et al. (2010), who detected differences in the levels of microbial metabolites in the urine of obese compared with lean individuals.

The first suggestion that changes in adiposity may influence gut microbiota was made in a study of patients undergoing intestinal bypass (Bjorneklett et al. 1981). More recently, bariatric surgery has been shown to alter gut ecology (Furet et al. 2010; Zhang et al. 2009) and improve glycemic control in type 2 diabetics (Ahima and Sabri 2011; Meijer et al. 2011). The mechanisms by which bariatric surgery corrects hyperinsulinemia are unknown (Reed et al. 2011; Tam et al. 2011), and information on the duration of this effect is not available (Schauer and Rubino 2011). Although it is not known whether the changes in microbial populations observed in patients after bypass surgery are directly related to changes in diabetes status, some studies suggest changes in bacterial populations may be related to obesity. Changes in specific bacterial populations after bariatric surgery include a reduction in methanogenic Archaea (Furet et al. 2010; Zhang et al. 2009). Zhang et al. (2009) hypothesized that in obese patients, methanogens could accelerate the fermentation of plant polysaccharides by lowering hydrogen gas production during fermentation, leading to higher acetate production and increased energy harvesting; however, that work was based on an extremely small sample size. Others have suggested that genetic factors may play an important role in determining the levels of gut methanogens (Hansen et al. 2011).

Experiments with germ-free or antibiotic-treated animals have yielded conflicting evidence regarding a role for gut microbes in the development of obesity. In some studies, germ-free mice demonstrated resistance to diet-induced obesity when fed a Western-type high sugar and fat diet (Backhed et al. 2004, 2007), whereas a more recent study using a different mouse line (C3H) found the opposite effect (Fleissner et al. 2010). In one mouse model, major differences were observed in the proportion of the different bacterial phyla in genetically obese mice and lean mice. Genetically obese ob/ob mice had a 50% reduction in the abundance of Bacteroidetes, and a proportional increase in Firmicutes (Ley et al. 2005). A metagenomic analysis revealed that the microbiome of obese mice had a higher percentage of genes associated with energy extraction than that of lean mice (Turnbaugh et al. 2006). Further work in the ob/ob mouse model demonstrated that this trait of increased energy extraction was transferable: weight gain and total body fat was higher in germ-free mice that received gut microbiota from obese mice than from lean mice. These differences were observed even though food consumption was the same (Turnbaugh et al. 2006).

In addition to their role in energy harvesting in the gut, microbiota may also affect obesity and diabetes risk via several other mechanisms including regulation of fat storage (Backhed et al. 2004), metabolic endotoxemia-induced inflammation (Cani and Delzenne 2007; Cani et al. 2008), and levels of satiety factors such as glucagon-like peptides and leptin (Cani et al. 2009; Ravussin et al. 2011; Sanz et al. 2010). For instance, C57BL/6J wild-type mice raised from birth with conventional gut microbiota had suppressed levels of fasting-induced adipocyte factor (FIAF) and a 42% higher body weight compared with germ-free mice. The broader relevance of this observation is unclear, since no differences were found in circulating FIAF levels of conventional C3H mice fed a Western or high-fat diet as compared with germ-free mice (Fleissner et al. 2010). Gut epithelial FIAF is a lipoprotein lipase inhibitor (LPL) and repressing its expression increases LPL activity and the storage of triglycerides in adipocytes (Backhed et al. 2004).

The metabolic inflammation hypothesis is based on the observation that mice fed a high-fat diet show changes in microbiota associated with increased intestinal permeability and go on to develop metabolic endotoxemia and inflammation. In ob/ob mice, gut microbiota composition affects plasma levels of endotoxin, presumably through altered gut permeability, which then leads to endotoxemia, inflammation, and metabolic changes that may influence the risk of obesity and diabetes (Cani et al. 2008). Cani et al. (2009) observed that prebiotics in humans can influence gut microbiota, which in turn affect the levels of gut satiety factors including glucagon-like peptide 1 and peptide YY. Obese-mouse studies characterizing diet and weight loss, and human studies characterizing how microbial communities are affected by diet, suggest complex interactions exist between diet, adiposity, gut microbiota, satiety hormones levels, and inflammation (Jumpertz et al. 2011; Muegge et al. 2011; Ravussin et al. 2011; Turnbaugh et al. 2009b, 2010).

Host genetic-gut ecology links may also affect immune function and the development of the suite of changes linked with metabolic syndrome (Vijay-Kumar et al. 2010). Mice lacking Toll-like receptor 5 (TLR5), which is important in immune system recognition of bacterial antigens in the colon, are hyperphagic with increased food consumption resulting in hyperlipidemia, hypertension, insulin resistance, and increased adipocity. Transferring gut microbiota from these TLR5 knock out mice to germ-free wild-type mice also resulted in hyperphagia and many of the same symptoms of metabolic syndrome. Unlike ob/ob mice that demonstrated phylum-level differences in microbiota, the TLR5 knock out and wild-type mice had similar proportions of Bacteroidetes and Firmicutes. However, marked differences in certain bacterial species in the TLR5 knock out mice were noted compared with wild-type mice. Surprisingly, Letran et al. (2011) did not observe basal inflammation or other metabolic changes in TLR5 knock out mice, although they did note a reduction in flagellin-specific CD4 T cells following *Salmonella* infection. The discrepancy between these reports, which used genetically identical mice, was suggested to lie in the different microbiota colonizing the mice at the different facilities. Thus, despite the apparently different outcome, these reports illustrate the importance of the gut microbiota and its complex interaction with the immune system.

Other studies suggest that microbiota may influence weight gain or loss and adiposity in humans. Ley et al. (2006) showed that obese humans had a lower Bacteroidetes to Firmicutes ratio than lean humans, but that this ratio increased with weight loss (Ley et al. 2006). Armougom et al. (2009) also found lower levels of Bacteriodetes in obese persons, although Arumugam et al. (2011) found no difference in this ratio.

Human studies also indicate weight gain during pregnancy can affect gut microbial populations and the incidence of obesity in offspring (Collado et al. 2008, 2010). Breast- versus formula-feeding practices, and vaginal versus cesarean section delivery also appear to affect the gut ecology of infants and may have relevance for obesity (Hallstrom et al. 2004; Musso et al. 2010; Penders et al. 2005, 2006). Another analysis of human microbiomes suggested that fecal microbiota composition in infants may predict later weight gain in children (Kalliomaki et al. 2008).

While debate is ongoing about the relevance of phylum-level differences in obese and lean individuals, research in these areas is still in its early stages. In order to thoroughly test the human adiposity-gut microbe hypothesis, additional carefully controlled experiments, as well as larger epidemiological studies, are needed.

## Gut Microbiome and Diabetes

In addition to the growing number of studies that suggest gut microbiota may affect the development of obesity, several studies suggest that the nature of the gut microbiota is linked to type 2 diabetes. This includes a study that found men with type 2 diabetes had significantly reduced levels of fecal Firmicutes, including Clostridia, compared with non-diabetic control subjects. Plasma glucose was positively correlated with both the ratios of Bacteroidetes to Firmicutes, and of the *Bacteroides–Prevotella* group to *Clostridium coccoides–Eubacteria rectale* group. In addition, the diabetic group also had more *Beta*-*proteobacteria* than non-diabetic controls. The authors suggested that the Bacteroidetes and Proteobacteria groups may affect diabetes risk via an endotoxin-induced inflammatory response, as both are gram-negative bacteria with lipid polysaccharide outer membranes (Larsen et al. 2010). Larsen et al. (2010) is the first study to show changes in microbial populations between type 2 diabetics compared with non-diabetics, but the study is based on a small number of subjects (*n* = 36), and these results need to be replicated in larger studies. It should also be noted that study subjects in both the diabetic and control groups had a wide range of BMIs.

Other researchers (Membrez et al. 2008) have investigated whether gut microbiota affects glycemic control and glucose tolerance using animal models of type 2 diabetes. In ob/ob mice, a 2-week treatment with antibiotics (norfloxacin and ampicillin) decreased gut levels of both aerobic and anaerobic bacteria. Antibiotic-treated ob/ob mice had significantly improved glucose tolerance; this was attributed to multiple factors including reduced liver triglycerides, increased liver glycogen, increased plasma adiponectin, and reduced plasma lipopolysaccarides (Membrez et al. 2008). The authors suggested that changes in the microbiota improved glucose tolerance via changes in metabolic and inflammatory pathways. Rabot et al. (2010) explored whether differences in insulin resistance and glycemic control exist between germ-free mice and mice with conventional gut microbes. They found that germ-free mice fed a high-fat diet consumed fewer calories, excreted more fecal lipids, and weighed less than conventional high-fat diet–fed mice. The germ-free mice also had reduced fasting and non-fasting insulinemia and improved glucose tolerance. Rabot et al. (2010) suggested these results support a role for gut microbiota in insulin sensitivity.

Relatively few studies have evaluated the role of microbes in type 1 diabetes. Vehik and Dabelea (2011) suggested that increased gut permeability (commonly called “leaky gut”) may affect the absorption of antigens that can attack and damage pancreatic beta cells. Bosi et al. (2006) observed increased gut permeability in human subjects with type 1 diabetes. Because gut microbes can affect intestinal permeability (Garcia-Lafuente et al. 2001), gut ecology may play a role in the development of type 1 diabetes (Neu et al. 2010). Another hypothesis by which microbes may cause type 1 diabetes is by producing bacterial toxins that can directly damage or affect the function of pancreatic beta cells. Myers et al. (2003) found that injecting the mice with *Streptomyces* toxin, bafilomycin A1, resulted in smaller pancreatic beta cells and impaired glucose tolerance. This *Streptomyces* toxin can be produced by soil microbes and subsequently infect commonly consumed root vegetables such as potatoes. Other microbial toxins, such as streptozotocin, have been used to induce diabetes in an experimental mouse model (Like and Rossini 1976). Little is known about other microbial toxins that may directly attack pancreatic beta cells and affect type 1 diabetes. Kootte et al. (2011) have speculated whether manipulating gut microbiota may have therapeutic benefits for treating patients with type 2 diabetes, including whether prebiotics, postbiotics, antibiotics or even microbial transplantation might have clinical significance.

## Obesogenic and Diabetogenic Environmental Chemicals

The possible role of chemical toxins in the rising rates of obesity world-wide was first proposed by Baillie-Hamilton (2002). Grün and Blumberg (2006, 2007) suggested that certain environmental pollutants, called “obesogens” can disrupt or interfere with the body’s homeostatic controls of adipogenesis, lipid metabolism, or energy balance. Adipose pathways involving nuclear receptors, such as the estrogen receptor (ER), retinoid X receptor (RXR), peroxisome proliferator-activated receptor-γ (PPAR-γ), and glucocorticoid receptors (GR), provided some of the first proposed molecular targets of environmental obesogens (Grün and Blumberg 2007). Casals-Casas and Desvergne (2011) have suggested that endocrine-disrupting chemicals that affect adipose and glucose-related pathways should be categorized into a subgroup called “metabolic disrupting chemicals.” Expanding the obesogen hypothesis, several researchers (Heindel and vom Saal 2009; Wolff et al. 2008) have proposed that environmental chemicals may act during critical windows of prepubertal and pubertal development to alter pathways involved in food intake, insulin sensitivity, lipid metabolism, and adipocyte development.

The level and strength of evidence (human vs. experimental animal or cell culture studies), the mechanism of action, and whether a dose–response effect or a low-dose effect (i.e., a U-shaped response curve) is observed, all vary by chemical. Although relatively few studies have examined whether environmental factors play a role in type 1 diabetes (Howard and Lee 2011), associations between the incidence of type 2 diabetes and exposure or use of a number of environmental chemicals is well supported in the human epidemiological literature for dichlorodiphenyldichloroethylene (DDE) (Codru et al. 2007; Cox et al. 2007; Lee et al. 2006; Rignell-Hydbom et al. 2009; Son et al. 2010; Turyk et al. 2009a, 2009b; Ukropec et al. 2010), hexachlorobenzene (HCB) (Codru et al. 2007; Ukropec et al. 2010), highly chlorinated polychlorinated biphenyls (PCBs) (Codru et al. 2007; Lee et al. 2006, 2010; Ukropec et al. 2010; Wang et al. 2008), dioxin (Henriksen et al. 1997; Kang et al. 2006; Michalek and Pavuk 2008), chlordane (Cox et al. 2007; Everett and Matheson 2010; Lee et al. 2006, 2007a, 2010, 2011; Son et al. 2010), and occupational exposure to agricultural insecticides and herbicides including chlordane, heptachlor, chlorpyrifos, diazinon, alachlor, cyanazine, and trichlorofon (Montgomery et al. 2008). Without mechanisms of action, however, it cannot yet be determined if all of the chemicals identified play a potential causal role, or if coexposures result in detecting some chemicals that do not have a biological effect on diabetes risk.

For other chemicals, there is a clearer picture of effects in human populations and mechanisms of action. Globally, high levels of arsenic in water supplies have been associated with increased incidence of type 2 diabetes (Chen et al. 2007; Navas-Acien et al. 2006; Rahman et al. 2009; Tseng 2007). Mechanistic studies suggest arsenic may impair insulin secretion from pancreatic beta cells and induce changes in gene expression affecting pancreatic insulin secretion and insulin resistance in peripheral tissues (Diaz-Villasenor et al. 2006, 2007). While a diabetogenic effect of another metal, cadmium, was noted in rats exposed neonatally (Merali and Singhal 1980), and suggestive evidence on fasting glucose levels in humans was reported (Schwartz et al. 2003), no mechanism of action has been identified.

Although strong evidence of a mechanism of action exists for other chemicals, few if any studies have documented whether past or current exposure levels in humans pose a risk. For example, strong mechanistic data support tributyltin as a developmental obesogen, especially for its action through nuclear receptor signaling. PPAR-γ is one of the key regulators of cell growth and differentiation of adipocytes. Tributyltin is an agonist for both PPAR-γ and the retinoid X receptor (RXR-α, -β and -γ) (Grün and Blumberg 2006; Grün et al. 2006), and tributyltin can sensitize human and mouse stromal stem cells to differentiate into adipocytes (Inadera and Shimomura 2005; Kirchner et al. 2010). Pubertal exposures in male mice cause increased body weight gain, hepatic steatosis, hyperinsulinemia, and hyperleptinemia (Zuo et al. 2011). However, the effect of environmental tributyltin exposure on related obesity disorders in human populations has not yet been investigated. More studies are needed to define the extent of human exposure from tributyltin’s use in anti-fouling marine paints and as a stabilizer in polyvinyl chloride plastics as well as its use in wallpaper, textiles, and floor coverings (Antizar-Ladislao 2008; Appel 2004; Kannan et al. 2010).

As discussed below, for several estrogenic environmental chemicals including bisphenol A (BPA), alkylphenols nonyl- and octylphenol, diethylstilbestrol (DES), and genistein, evidence from animal and tissue culture models indicate that these environmental estrogens affect a variety of other receptor-mediated, cellular, and molecular targets linked to adipose and/or glucose metabolism. GR signaling is central to adipocyte differentiation. For example, using the 3T3-L1 preadipocyte cell line, Sargis et al. (2010) demonstrated that BPA stimulated GR and increased lipid accumulation in the differentiating adipocytes. BPA can also modulate glucose transport in mouse 3Y3-F442A adipocytes, enhancing the level of a key glucose transport protein GLUT4 (Sakurai et al. 2004). Other studies have shown that BPA can suppress the release of adiponectin (which can affect insulin sensitivity and resistance) from human adipocytes or adipose explants (Hugo et al. 2008).

Evidence from animal studies on the effects of early-life BPA exposure on obesity is not consistent; effects appear to depend on route of exposure, sex, and species (Miyawaki et al. 2007; Rubin et al. 2001; Ryan et al. 2010). Human studies have not provided strong evidence of an effect on obesity for current levels of human BPA exposure (Lang et al. 2008; Melzer et al. 2010).

Using 3T3-L1 adipocytes, researchers have found that the alkylphenols octylphenol and nonylphenol up-regulate the expression of the resisten gene, which affects insulin resistance and decreases adipocyte differentiation. Male rats treated with octylphenol show increased serum levels of glucose (Lee et al. 2008). Although nonylphenol has been widely detected in human adipose tissue, a positive relationship between the measures of obesity such as BMI and adipose levels for this environmental estrogen has not been shown (Lopez-Espinosa et al. 2009).

Neonatal exposure of mice to the nonsteroidal estrogen DES results in an initial weight loss followed by an increase in body fat by 2 months of age (Newbold 2010). DES exposure increases serum leptin and triglycerides levels and changes the expression of several genes involved in fat distribution (Newbold et al. 2007). Cohort studies have not yet determined whether there is a higher incidence of obesity in DES mothers, or in their children exposed to DES *in utero*, even though this compound has been found to have multigenerational effects on other end points such as female cervical cancer and male urogenital malformations (Palmer et al. 2009; Troisi et al. 2007).

For the phytoestrogen genistein, effects on gene expression of adipose-related factors, including induction of phospholipase A2 group 7 and phospholipid transfer protein genes, were seen at low, but not high, doses in a mouse study (Penza et al. 2006). This suggests that for some environmental chemicals, especially those with hormonal action, low-dose effects need to be examined rather than relying on traditional high-dose response effects. U-shaped response curves have been reported for other environmental chemicals, including some congeners of polybrominated diphenyl ether (PBDE) flame retardants including PBDE-153, and certain PCBs (Lee et al. 2007b, 2011; Lim et al. 2008). The U-shaped response curves suggest that future research is needed to determine if other chemicals have low-dose effects on metabolic syndrome, obesity, and diabetes.

The effects of individual chemicals on obesity and diabetes cannot be generalized to entire classes of chemicals. For instance, type 2 diabetes–related effects of PCBs and brominated flame retardants [polybrominated biphenyls (PBBs), and PBDEs] appear to be more closely associated with highly halogenated forms of these chemicals (Everett et al. 2007; Lee et al. 2010; Lim et al. 2008). Only certain types or metabolites of phthalates appear to be associated with obesity or diabetes in humans (Hatch et al. 2008). In rodent studies, diisobutylphthalate shows some evidence of affecting obesity via PPAR pathways (Boberg et al. 2008), but evidence for other phthalates, including diethylhexyl phthalate, is less consistent (Casals-Casas et al. 2008; Feige et al. 2010).

For other chemicals such as the PPAR agonist perfluorooctanoic acid (PFOA), evidence of an association with obesity and diabetes is emerging but inconsistent. Although there is some evidence of a higher incidence of diabetes in persons who are occupationally exposed to PFOA (Lundin et al. 2009), a large-scale cross-sectional epidemiological study did not observe a relationship between PFOA levels and type 2 diabetes or fasting glucose levels (MacNeil et al. 2009). Rodent studies indicate that PFOA can be transmitted from the dam to pup during lactation (Fenton et al. 2009), and there is some evidence of PFOA being a developmental obesogen in mice (Hines et al. 2009), but studies in rats have not indicated an effect of early-life PFOA exposure on plasma insulin or leptin levels (Boberg et al. 2008).

## Disposition of Environmental Chemicals

In addition to the host–microbe interactions and the direct effects of the chemicals discussed above, we suggest that microbes may affect obesity and diabetes by altering the ADME of environmental chemicals. Microbially mediated effects on ADME could include the direct activation of chemicals (Van de Wiele et al. 2005, 2010; Wallace et al. 2010), production of microbial metabolites that compete for limited host biotransformation capacity (Clayton et al. 2009; Wallace et al. 2010), alteration of host biotransformation enzyme activities (Claus et al. 2011; Meinl et al. 2009), changes in enterohepatic circulation (Meijer et al. 2006), or altered bioavailability of environmental chemicals and/or antioxidants from food (Kemperman et al. 2010; Lhoste et al. 2003; Oishi et al. 2008; van Duynhoven et al. 2010). Increased bioavailability may also result from changes in gut motility and barrier function. Although evidence indicates that the ADME of environmental chemicals may be affected by many of these microbial-mediated pathways, no studies have evaluated how the ADME of obesogenic or diabetogenic chemicals are affected by variations in the human microbiome. Thus, there is a need to determine the effect of microbes on the bioavailability of environmental chemicals, and the direct biotransformation of persistent organic pollutants (Dean and Ma 2007; Possemiers et al. 2009).

Using an *in vitro* model that simulates the human intestinal microbial system (biota cultured from human feces), Van de Wiele et al. (2005) demonstrated that colonic microbiota were capable of transforming polyaromatic hydrocarbons (PAHs) to the bioactive estrogenic metabolites 1-hydroxypyrene and 7-hydroxybenzo[*a*]pyrene, whereas stomach and small intestine digests of the PAH did not produce estrogenic metabolites. This finding suggests that colonic microbes can biotransform parent compounds directly into active metabolites. Gut microbes were found to thiolate and methylate arsenic in both human and mouse models (Pinyayev et al. 2011; Van de Wiele et al. 2010). Exposure to high levels of arsenic have been associated with both an increased risk of bladder cancer and a higher incidence of diabetes in persons living in areas with contaminated water supplies and/or seafood (Chen et al. 2007; Coronado-Gonzalez et al. 2007; Kim and Lee 2011; Navas-Acien et al. 2006; Rahman et al. 2009; Yen et al. 2007). Although these microbial model systems suggest the capacity for biotransformation of environmental chemicals in the gut, especially the colon, their impact on systemic pollutant levels is not known. The extent of biotransformation variation by the gut microbes, so called “presystemic metabolism” (Grundmann 2010) of different individuals is also not known. Despite phylogenetic diversity, the implied functional metabolic redundancy observed in the gut metagenomes of individual twins (Turnbaugh et al. 2009a) raises the question as to whether or not important differences exist between the enzymatic capacities of individuals. Although no large-scale functional studies have been done to characterize the interindividual variation in gut microbe enzymatic capacity, the available human and rodent data suggest that variations in gut microbiota affect environmental chemical disposition (McBain and MacFarlane 1998; Rowland et al. 1985). This has been indirectly established in the area of colon cancer where variation in fecal enzyme activities has been found to correlate with cancer risk (Rowland 2009).

The pharmacology literature provides valuable evidence demonstrating how chemical fate can be affected by variability in the host microbiome (Clayton et al. 2009; Sousa et al. 2008; Wallace et al. 2010; Wilson 2009). For example, a 2008 review identified 30 drugs (including chloramphenicol) that can be metabolized by gut microbiota whose metabolism shows considerable interindiviual variability depending on the presence or absence of specific bacteria genera (Sousa et al. 2008). In addition, studies have shown that variations in gut microbiota can affect the metabolism of commonly used over-the-counter drugs such as acetaminophen (Clayton et al. 2009) and that limiting gut microbial metabolism of chemotherapy drugs can reduce drug toxicity (Wallace et al. 2010).

Recent evidence suggests that acetaminophen’s metabolism and toxicity are affected by individual variations in the gut microbiome (Clayton et al. 2009). Some gut bacteria, including *Clostridium difficle*, metabolize tyrosine to *p*-cresol, which can compete with acetaminophen for sulfonation in the gut. In individuals whose gut bacteria produce high levels of *p*-cresol, less acetaminophen undergoes sulfonation because of competition with *p*-cresol and more is glucuronidated. Researchers found that the ratio of sulfonated to glucuronidated *p*-cresol in the urine was predictive of acetaminophen toxicity. The same markers are likely relevant to metabolism of other compounds that rely on these pathways for detoxification (Clayton et al. 2009), including many that have been suggested to be environmental obesogens or diabetogens [see Supplemental Material, Table 1 (http://dx.doi.org/10.1289/ehp.1104204)].

Another example of gut ecology–pharmaceutical interaction is the metabolism of the chemotherapeutic prodrug CPT-11. Upon administration, CPT-11 is first activated by carboxyesterases in the liver to yield toxic SN38, which in turn is glucuronidated by uridine diphosphate (UDP)-glucuronosyltransferase to nontoxic SN38G. SN38G is excreted into the bile and returned to the gut where β-glucuronidases from commensal gut bacteria remove the glucuronide. This reactivates the drug in the gut, which can in turn cause bloody diarrhea, limiting the dose that can be used in chemotherapy. To circumvent the unintended intestinal toxicity of SN38, researchers developed an inhibitor of microbial glucuronidase that was not toxic to gut microbes, but which prevented the metabolism of SN38G and thereby increased mouse tolerance to CPT-11 (Wallace et al. 2010). Microbial glucuronidase activity also has been shown to be important in activating foodborne procarcinogens in the gut (Humblot et al. 2007), further illustrating the role of this enzyme in chemical metabolism.

The above examples underscore the reasons recent reviews in the pharmacology literature have articulated the need for future drug development to include an integrated assessment of host and environmental factors, including gut microbes that affect drug disposition (Grundmann 2010; Sousa et al. 2008; Wilson 2009; Wilson and Nicholson 2009). This topic has received little attention in the toxicology literature (Possemiers et al. 2009). In this regard, pharmacometabolomics appears to be an important emerging tool for investigating how gut ecology may affect the fate of chemical toxicants and their contribution to diabetes and obesity risk. Given the established functional differences in the microbiomes of obese and lean humans (Arumugam et al. 2011), microbes may be an important source of variation in the ADME profile of obesogenic and diabetogenic chemicals and deserve increased attention.

This is especially important in light of evidence in animal models that suggest changes in gut microbiota not only affect levels of gut metabolic enzymes, but levels of hepatic enzymes as well (Claus et al. 2011; Meinl et al. 2009; Reddy et al. 1973). The ability of gut microbiota to affect levels of hepatic enzymes was initially demonstrated for carbohydrate-metabolizing enzymes. Compared with germ-free rats, conventional rats displayed a significantly higher activity of hepatic glucose-6-phosphate dehydrogenase (Reddy et al. 1973). Limited recent evidence indicates that certain phase I enzymes also can be influenced by gut microbiota. The expression and level of P450 enzymes Cyp3a11 and Cyp2c29 were significantly higher in the livers of mice with conventional gut microbiota compared with germ-free control mice (Claus et al. 2011).

Several hepatic and gut phase II enzymes also have been compared in germ-free and germ-free rats “reassociated” with conventional microbiota (Meinl et al. 2009). These include glutathione transferases, glutathione peroxidase, epoxide hydrolases, acetyltransferases, and sulfotransferases. Levels of isoenzymes were compared in the liver, small intestine, cecum, and colon of germ-free and reassociated control rats. In most cases, germ-free rats had higher levels of colonic phase I and II enzymes than control rats with conventional microbiota, although there was no effect of germ-free status on the levels of enzymes in the small intestine. Differences were, however, observed in the level of liver enzymes in germ-free rats compared with reassociated rats of both sexes, with elevations in sulfotransferases. Hepatic epoxide hydrolase was elevated in germ-free rats as well, but only in females (Meinl et al. 2009).

Levels of hepatic biotransformation enzymes can also be affected by diet-induced changes in gut microbes (Treptow-van Lishaut et al. 1999). Levels of glutathione *S*-transferase-π (the predominant GST isoenzyme) were lower in colon cells of germ-free rats compared with colon cells of rats with conventional microbiota. The levels of this enzyme increased 3-fold when the diet of rats with conventional microbiota was changed from a highly digestible maize starch to a poorly digestible high-amylose maize starch. The authors suggested that amylose was fermented in the colon, and may have yielded short-chain fatty acids such as n-butyrate, which may have induced GST (Treptow-van Lishaut et al. 1999). Changes in gut microbiota composition were not measured with the dietary change, so it is unknown to what extent gut microbiota may have affected induction of GST directly; however, this report does suggest that changes in gut microbial activity (fermentation) correlate with changes in this phase II enzyme that plays an important role in cellular detoxification (Di Pietro et al. 2010). The potential for diet/microbe-enhanced induction of detoxification capacity demonstrated in the colon of animal models contributes to interest in the potential detoxification and anticancer effects of both pre- and probiotics; however, further human studies are required (Genuis 2011).

Some evidence from laboratory animal studies indicates that the polyphenols quercetin and catechin may influence liver or gut levels of phase II enzymes (Lhoste et al. 2003; Wiegand et al. 2009) and that gut microbes play a role in polyphenol-mediated enzyme induction (Lhoste et al. 2003). Few studies have looked at the levels or activity of phase II biotransformation enzymes in the human gut (Peters et al. 1991; Teubner et al. 2007). Little is known concerning to what extent activities of these biotransformation enzymes are affected by the wide variations in the human microbiome. Hence, we have virtually no information on how variations in gut ecology affect human ADME capacity with respect to environmental chemicals. Understanding how other dietary components, including polyphenols, might modify gut microbial populations and levels of phase I and II enzymes, may yield important information relevant to interindividual variation in chemical metabolism (Kemperman et al. 2010; van Duynhoven et al. 2010).

Another area where little information exists is whether the enterohepatic circulation of environmental chemicals is affected by variation in gut microbial populations. Since many environmental toxicants undergo phase II metabolism [see Supplemental Material, Table 1 (http://dx.doi.org/10.1289/ehp.1104204)], those that are excreted in the bile may be further metabolized by the enzymes of gut microbiota, including glucuronidases, leading to enterohepatic circulation and increased residence time in the body (Humblot et al. 2007). The importance of this process has been demonstrated by administration of nonabsorbable fat (i.e., sucrose polyester), which decreases enterohepatic circulation and increases fecal fat excretion of the flame retardant PBDE-47 (Meijer et al. 2006). To what extent enterohepatic circulation of lipophillic persistent pollutants, including obesogenic or diabetogenic chemicals, can be influenced by variations in gut microbial populations is unclear; however, the limited information available suggests that the variability in the activity levels of relevant gut microbe enzymes may be quite high, especially for β-glucuroronidase (Rowland et al. 1986). Given the importance of microbial β-glucuronidases in enterohepatic cycling, and the enrichment of related genes (e.g., those encoding hydrolases) in obesity-associated microbiomes (Turnbaugh et al. 2009a), it is important that we understand interindividual differences in enterohepatic cycling and how they may affect the risk of obesity or diabetes.

## Conclusions and Future Research Needs

Microbial populations and/or metabolic capacities are known to differ in obese and lean subjects (and in type 2 diabetes), yet we know surprisingly little about the effect of these differences on the body burden of obesogenic and diabetogenic chemicals. The ability to characterize and manipulate microbial populations in gnotobiotic mice, however, including humanizing of the rodent gut (Goodman and Gordon 2010), provide us with an unparalleled opportunity to begin exploring the impact of gut microbe variability on the disposition of environmental chemicals in humans. Future research in this area should quantify how interindividual variations in gut microbiota affect the body burden of environmental chemicals by altering *a*) these chemicals directly, *b*) the level and activity of host phase I and II enzymes, *c*) enterohepatic circulation of environmental chemicals, *d*) depletion of host detoxification capacity, and *e*) alterations of gut barrier function. Studies should also identify biomarkers that are predictive of impaired obesogenic and diabetogenic chemical absorption, distribution, metabolism, and excretion and assess the interaction between microbiota and developmental obesogens, including intergenerational effects. This approach will shed light on how variations in gut ecology affect the metabolism of obesogenic and diabetogenic chemicals and lead to more personalized approaches in the treatment and prevention of obesity and diabetes.

## Supplemental Material

(201 KB) PDF
