# Supplementary material for: Do Interactions Between Gut Ecology and Environmental Chemicals Contribute to Obesity and Diabetes?
Source: Environ Health Perspect. 2011 Oct 31;120(3):332–9. doi: 10.1289/ehp.1104204 (PMC3295356; doi:10.1289/ehp.1104204)
Supplement: (201 KB) PDF [file ehp.1104204.s001.pdf]

Supplemental Materials

Do Interactions Between Gut Ecology and Environmental Chemicals Contribute to Obesity and Diabetes?

Suzanne M. Snedeker[1,2] and Anthony G. Hay[1,3]

[1]Department of Microbiology and the Institute for Comparative and Environmental Toxicology, Cornell University, Ithaca, New York, 14853, USA

[2] Department of Food Science, Cornell University, Ithaca, New York, 14853, USA

[3] Address correspondence to: A.G. Hay, Department of Microbiology, B75C Wing Hall, Wing Drive, Cornell University, Ithaca, NY 14853, USA. E-mail: agh5@cornell.edu

Supplemental Material, Table 1. Phase II metabolism of obesogenic/diabetogenic chemicals[a]

	Enterohepatic cycling	Phase II conjugates			References
**Non-pesticide persistent organic pollutants**		Glucuronide	Sulfate	Glutathione	
Polyfluorinated alkanoic acids	AE[a]				NA
Polybrominated flame retardants	y[a]	y	y		(Schauer et al. 2006)
Polychlorinated biphenyls	y	y	y	y	(Redgrave et al. 2005)
Polychlorinated dibenzodioxins	y	y			(Hakk et al. 2001)
Polychlorinated dibenzofurans	y	y	y	y	(Burka et al. 1990)
**Pesticides**					
Alachlor	y			y	(Heydens et al. 1999)
Aldrin	y	y			(ATSDR 2002a)
Amitraz	y	y	y		(Hornish 1984)
Chlordane	EA[a]	y			(ATSDR 1994)
Chlorpyrifos	y	y			(ATSDR 1997)
Chlorpyrifos methyl	AE				(ATSDR 1997)
Cyanazine	AE			y	(Crayford and Hutson 1972)
DDD	y	y			(ATSDR 2002b)
DDE	y	y			(ATSDR 2002b)
DDT (o,p’ and p,p’)	y	y			(ATSDR 2002b)
Diazinon	AE				(ATSDR 2008)
Dieldrin	y	y			(ATSDR 2002a)
Endrin	y	y			(ATSDR 2002a)
Heptachlor	y	y	y	y	(ATSDR 2007)
Hexachlorobenzene	y	y	y	y	(ATSDR 2002c)
Hexachlorocyclohexane-γ & β	y	y	y	y	(ATSDR 2005)
Mirex	AE				(ATSDR 1995)
Trans-nonachlor	AE				(Tashiro and Matsumura 1978)
Trichlorofon	y	y			(Vale 1998)
Vacor	AE				
**Metals**					
Arsenic[b]	y			y	(Klaassen 1974)
Cadmium	y			y	(Cherian and Vostal 1977)
Lead	AE				
Mercury[b]	y			y	(Clarkson and Magos 2006)
Tributyl tin	y				(Iwai et al. 1982)
**Other chemicals**					
Bisphenol A	y	y	y		(Ginsberg and Rice 2009)
Diethylstilbestrol	y	y	y		(Metzler 1981)
Genistein	y	y			(Shelnutt et al. 2002)
Nicotine	AE				(Hukkanen et al. 2005)
Nonylphenol	y	y			(Green et al. 2003)
Octylphenol	y	y			(Upmeier et al. 1999)
Phthalates	y	y			(Seckin et al. 2009)
**Totals**	**27/37**	**23/37**	**9/37**	**10/37**	

Supplemental Material, Table 1. Phase II metabolism of obesogenic/diabetogenic chemicals[a]

Abbreviations: 1,1-*bis*-(4-chlorophenyl)-2,2-dichloroethene (DDD); 1,1-*bis*-(4-chlorophenyl)-2,2-dichloroethene (DDE); 1,1-*bis*-(4-chlorophenyl)-2,2,2-trichloroethane (DDT).

Footnotes:

[a] The information in this table indicates if evidence was found (y=yes, AE= absence of evidence, EA=evidence of absence) that obesogenic/diobetogenic environmental chemicals undergo enterohepatic cycling. The nature of the phase II conjugates is indicated, (either glucuronide, sulfate, or glutathione) and relevant, since these can be further metabolized by gut microbiota. It is likely that interindividual variation in gut microbiota will play an important role in the bioavailability of these environmental chemicals and may result in increased exposure, thereby increasing the risk of obesity and diabetes.

[b] Both arsenic (As) and mercury (Hg) can be methylated by gut biota, while As has also been shown to be thiolated (Van de Wiele et al. 2010).

References for Supplemental Material

Table 1. Phase II metabolism of obesogenic/diabetogenic chemicals

ATSDR. 1994. Toxicological profile for chlordane. Atlanta:U.S. Dept. of Health and Human Services, Public Health Service, Agency for Toxic Substances and Disease Registry.

ATSDR. 1995. Toxicological profile for mirex and chlordecone. Atlanta:U.S. Dept. of Health and Human Services, Public Health Service, Agency for Toxic Substances and Disease Registry.

ATSDR. 1997. Toxicological profile for chlorpyrifos. Atlanta:U.S. Dept. of Health and Human Services, Public Health Service, Agency for Toxic Substances and Disease Registry.

ATSDR. 2002a. Toxicological profile for aldrin/dieldrin.U.S. Dept. of Health and Human Services, Public Health Service, Agency for Toxic Substances and Disease Registry.

ATSDR. 2002b. Toxicological profile for 4,4'DDT, 4,4'DDE, 4,4' DDD (update). Atlanta:U.S. Dept. of Health and Human Services, Public Health Service, Agency for Toxic Substances and Disease Registry.

ATSDR. 2002c. Toxicological profile for hexachlorobenzene. Atlanta:U.S. Dept. of Health and Human Services, Public Health Service, Agency for Toxic Substances and Disease Registry.

ATSDR. 2005. Toxicological profile for hexachlorocyclohexane. Atlanta:U.S. Dept. of Health and Human Services, Public Health Service, Agency for Toxic Substances and Disease Registry.

ATSDR. 2007. Toxicological profile for heptachlor and heptachlor epoxide. Atlanta:U.S. Dept. of Health and Human Services, Public Health Service, Agency for Toxic Substances and Disease Registry.

ATSDR. 2008. Toxicological profile for diazinon. Atlanta:U.S. Dept. of Health and Human Services, Public Health Service, Agency for Toxic Substances and Disease Registry.

Burka LT, McGown SR, Tomer KB. 1990. Identification of the biliary metabolites of 2,3,7,8-tetrachlorodibenzofuran in the rat. Chemosphere 21(10-11):1231-1242.

Cherian MG, Vostal JJ. 1977. Biliary excretion of cadmium in rat. I. Dose-dependent Biliary excretion and the form of cadmium in the bile. J Toxicol Environ Health 2(4):945 - 954.

Clarkson TW, Magos L. 2006. The toxicology of mercury and Its chemical compounds. Crit Rev Toxicol 36(8):609-662.

Crayford JV, Hutson DH. 1972. The metabolism of the herbicide, 2-chloro-4-(ethylamino)-6-(1-cyano-1-methylethylamino)-S-triazine in the rat. Pest Biochem Physiol 2(3):295-307.

Ginsberg G, Rice DC. 2009. Does rapid metabolism ensure negligible risk from bisphenol A? Environ Health Perspect 117(11):1639-1643.

Green T, Swain C, Van Miller JP, Joiner RL. 2003. Absorption, bioavailability, and metabolism of para-nonylphenol in the rat. Regul Toxicol Pharmacol 38(1):43-51.

Hakk H, Larsen G, Feil V. 2001. Tissue distribution, excretion, and metabolism of 1,2,7,8-tetrachlorodibenzo-p-dioxin in the rat. Chemosphere 42(8):975-983.

Heydens WF, Wilson AG, Kier LD, Lau H, Thake DC, Martens MA. 1999. An evaluation of the carcinogenic potential of the herbicide alachlor to man. Hum Exp Toxicol 18(6):363-391.

Hornish RE. 1984. Metabolism of four thiobis(formamidine) insecticides in the mouse and the rat. J Agricult Food Chem 32(1):114-119.

Hukkanen J, Jacob P, Benowitz NL. 2005. Metabolism and disposition kinetics of nicotine. Pharmacol Rev 57(1):79-115.

Iwai H, Wada O, Arakawa Y, Ono T. 1982. Intestinal uptake site, enterohepatic circulation, and excretion of tetra-and trialkyltin compounds in mammals. J Toxicol Environ Health 9(1):41-49.

Klaassen CD. 1974. Biliary excretion of arsenic in rats, rabbits, and dogs. Toxicol Appl Pharmacol 29(3):447-457.

Metzler M. 1981. The metabolism of diethylstilbestrol. CRC Crit Rev Biochem 10(3):171-212.

Redgrave TG, Wallace P, Jandacek RJ, Tso P. 2005. Treatment with a dietary fat substitute decreased Arochlor 1254 contamination in an obese diabetic male. J Nutr Biochem 16(6):383-384.

Schauer UMD, Völkel W, Dekant W. 2006. Toxicokinetics of tetrabromobisphenol A in humans and rats after oral administration. Toxicol Sci 91(1):49-58.

Seckin E, Fromme H, Völkel W. 2009. Determination of total and free mono-n-butyl phthalate in human urine samples after medication of a di-n-butyl phthalate containing capsule. Toxicol Lett 188(1):33-37.

Shelnutt SR, Cimino CO, Wiggins P, A., Ronis MJJ, Badger TM. 2002. Pharmacokinetics of the glucuronide and sulfate conjugates of genistein and daidzein in men and women after consumption of a soy beverage. AJCN 76(3):588-594.

Tashiro S, Matsumura F. 1978. Metabolism of trans nonachlor and related chlordane components in rat and man. Arch Environ Contam Toxicol 7(1):113-127.

Upmeier A, Degen GH, Schuhmacher US, Certa H, Bolt HM. 1999. Toxicokinetics of p-tert-octylphenol in female DA/Han rats after single i.v. and oral application. ArchToxicol 73(4):217-222.

Vale JA. 1998. Toxicokinetic and toxicodynamic aspects of organophosphorus (OP) insecticide poisoning. Toxicol Lett 102-103:649-652.

Van de Wiele T, Gallawa CM, Kubachka KM, Creed JT, Basta N, Dayton EA, et al. 2010. Arsenic metabolism by human gut microbiota upon *in vitro* digestion of contaminated soils. Environ Health Perspect 118(7):1004-1009.
